# Dr. E. G. Munn, the Rochester Ophthalmologist, as One of the Earliest Specialists in America; Together with the Present-Day Rochester Ideas on Specialism and Medico-Legal Expert Testimony

**Published:** 1912-12

**Authors:** C W. Hennington

**Affiliations:** Rochester, N. Y.


### Dr. E. G. Munn, the Rochester Ophthalmologist, As One of the Earliest Specialists in America; Together with the Present-day Rochester Ideas on Specialism and Medico-Legal Expert Testimony

By DR. C W. HENNINGTON
Rochester, N. Y.

DR. Edwin George Munn was born in Munson, Mass., in 1804, either on April 7th or May 8th. He sprang from early colonial ancestry. While he was still a child, the family removed to LeRoy, N. Y., where he grew up. Having completed his literary education, he began the study of medicine with Dr. Stephen O. Almy of that place. Later he went to Fairfield and to Philadelphia to complete his medical study.

Dr. Munn early became interested in diseases of the eye, in fact while he was still with Dr. Almy, and in this manner, as used to be related by our late Dr. Edward Mott Moore. It seems that there were many cases of sore eyes in that new country at that early time and when Dr. Almy told such persons that he did not know how to help them, they would turn to the young student, our Dr. Munn, and say; "Ed, for God's sake, try to help us," and in this way "Ed" became interested in the eye

In 1828 Dr. Munn, then twenty-four years old, located in practice in Scottsville, N. Y., and continued his special interest in the eye. He lived in Scottsville only nine years and during this time his reputation spread throughout Western New York. It is astonishing to realize from what distances patients traveled in order to consult him, even during this period of his early residence in Scottsville. A Mr. W. H. Abell, long since dead, who was for many years the President of the Western Elevating Association of Buffalo, used to narrate how he had taken his sister, a girl of twelve or fifteen years, in a cutter all the way from Fredonia, N. Y., to Scottsville, N. Y., probably fully one hundred and thirty miles distant, hoping that Dr. Munn would be able to restore her sight. She had been born blind, evidently congenital cataract, and later obtained her sight. From her descriptions other details of Dr. Munn's life during this time that she was in his care are known to us. "After practicing a few years in Scottsville," Dr. Edward Mott Moore says, "he found himself overwhelmed with the duties of an oculist," and, "in 1837 he removed to Rochester and gave himself up to the duties of ophthalmology." At the time of his removal to Rochester, Dr. Munn was but thirty-three years old. He practiced in Rochester exactly ten years until his death in 1847 on December 12th, thus having reached the age of only forty-three years. Dr. Moore says: "It is doubtful if, during the ten years of his residence here any other man had so many patients and gathered from as large a territory. As the people generally were poor, much of this great following came from his generous disposition and as long as there was money in his purse he was very apt to pay the board bills of his patients and their passage home after their recovery." At that time there was no one in New York City who knew much about the eye and many persons came from there to Rochester to be treated. From a study of Dr. Munn's entries in his records it appears that patients came from Arkansas, Missouri, Illinois, Michigan, and even some of the southern states to consult him. It is said that at times nearly a hundred people waited at his office. He had purchased a home just outside of Rochester in Gates to which he might retire for rest and seclusion at the end of each day's labor.

It has been difficult to obtain facts that indicate Dr. Munn's exact professional attainments. Dr. Edward Mott Moore, who we consider the Nestor of the profession in our part of the country regarded him as a man of brilliant attainments and great originality. He spoke of his dissecting and experimenting and of a great amount of research work in this new field. Thus we have the truly marvelous history of a short professional life.

A few personal facts might be added. While yet in Scottsville in 1834, Dr. Munn married Aristine Pixley who was considerably younger than he and who still survives at the advanced age of ninety-five years. They had three children, of whom one, the youngest, is Dr. John P. Munn, of New York.

As we study the condition of ophthalmology in the United States in Munn's time, our appreciation of his life is even greater than when it is viewed without the back ground of the period.

When Dr. Munn entered practice in the little Village of Scottsville in 1828 and soon found himself able to devote his efforts to ophthalmology alone, there was probably but one other ophthalmologist in all the United State and that was the celebrated Dr. George Frick (1793-1870) of Baltimore. At that time Dr. Frick was still a young man, though about ten years the senior of Dr. Munn. He had begun six years before, in 1822, to give lectures on diseases of the eye in the Maryland General Hospital and was devoting himself to his specialty, having also published his "Treatise on Diseases of the Eye," in 1823.

To be sure, the first Eye Infirmaries in this country had been started in New London, 1817, New York, 1820, Philadelphia, 1821, Baltimore, 1823, but the men who founded and served at these institutions were general physicians and surgeons who had acquired some special knowledge of ophthalmology. Only a few of these earlier men like Frick could be said to actually devote themselves to that specialty.

In Dr. Roswell Park's History of Medicine we read: "As illustrating how little our present specialties were then separated, it is worth while to remark that Dr. Edward Delafield, who in 1826 was Professor of Obstetrics and Diseases of Women and Children in the College of Physicians and Surgeons, New York, delivered at the same time a special course of lectures upon Diseases of the Eye!"

Dr. George Frick of Baltimore, deserves the title of the first ophthalmologist in the United States. Dr. Munn undoubtedly should be placed next and then in point of time should follow such men as Squier Littell and Isaac Hays. Following them comes Ezra Dyer, George Strawbridge and Elkanah Williams of Cincinnati, who returned from abroad in 1855 with the new ophthalmoscope and of whom it is said: "His specialty was quite an innovation at that time (1855) ; the operative part of ophthalmology was within the province of the surgeon and the ordinary diseases of the eye were treated by the general practitioner.

It makes us marvel that this achievement of Munn's was not more greatly appreciated and that historical articles and books fail to mention him. It has remained for us to fix his claim as a contemporary of the justly esteemed George Frick, and undoubtedly the second opthalmologist in America.

Thus a man who deserves national fame has failed to receive suitable recognition even by his own community. He was honored and esteemed, but not to the degree that we now realize should have been his. We have another illustration of the ancient adage of the prophet without honor in his own country. It is a psychologic fact that it is difficult to estimate either the man or the idea that is near to us. We fail to get the proper perspective. Probably Dr. Munn himself also failed to value his life at its true worth.

With this text I wish to bring to your attention the Rochester idea of specialism and medico-legal expert testimony. We are proud that it should be called the Rochester idea for to Dr. Seelye W. Little belongs the credit of originating and advocating this innovation and of putting it to an actual test during his present term as President of our County Society. It would be unfortunate indeed if, because of lack of appreciation of the full significance of this movement, we failed to develop the entire plan as he has repeatedly outlined it. The purpose of this paper is to challenge you to follow out the details of the idea and to make the obvious applications of practical value.

It seems wise at this point to refer to Dr. Little's concise statement in the BUFFALO MEDICAL JOURNAL of May, 1912, under the title of "A New Departure in the Medical Expert Matter."

As is well known to us our Monroe County Society at the May, 1912, meeting did adopt a list of thirteen specialists in psychiatry of which seven are members of the staff at the Rochester State Hospital, and six are practicing physicians in the city. This list was sent to every Court and to every Lawyer in the County. Only a half year has elapsed, but the comments and discussion that was provoked proved valuable. The actual result in practice will be watched with much interest.

For the welfare of the profession it would seem desirable to apply the innovation very gradually to the other specialties and only after most careful consideration in each case. The definite specialties such as Ophthalmology, Roentgenology and perhaps some others might be shortly included. Whatever lists are published should be constantly supervised and kept accurate. We might be wise to empower the Board of Censors to officially investigate any charges of alleged unethical conduct on the part of those on these lists.

That this medical expert testimony matter is a subject of very considerable interest and involves the standing and good name of the profession is indicated by a thorough editorial in the *New York State Journal of Medicine* for this September, 1912, in which the statutes of all the states in the Union are reviewed. The difficulties of solving the problem by legislative enactment are commented upon at length. The editor evidently has not considered our Rochester idea in this problem, and fails to appreciate that possibly there may be other methods of giving authority to medical testimony than by legislation.

In this paper, however, I wish to call your attention to the indirect advantages that will be felt by the profession itself and by its individual members. This phase of the matter has not been dwelled upon in so far as I know and it impresses me as of prime importance. It may be that these subtle indirect effects will ultimately outweigh the more obvious effects. The first indirect effect will be to emphasize the unity of interest of the whole profession. It will cause the individual to make his acts conform more closely to the ideals of our body. Ethically it will tend to improvement in the individuals and at large.

The second indirect effect it seems to me will be intellectual. Even if there be lists in but one or a few branches of medicine, the recognition of the principle involved will react very favorably on all the departments of medicine. There will be placed a premium upon more intensive effort for perfection in one branch in contrast to the present extensive effort to grasp and remain cognizant with the advance in the whole field.

Another indirect effect will be to tend toward limiting the number of specialists for new men about to announce their entrance upon a given specialty will realize that their preparation and experience and ability will be subjected to careful scrutiny in case it should be proposed to establish a list of those engaged in that specialty. It is obvious that this result will be attained without the establishment of such a list, but merely because that contingency exists. In fact the more gradually we attack the matter, the greater will be the advantage to the profession in compelling the most thorough training of our specialists. Another indirect effect will be the immediate disappearance of such absurd combinations of specialties as are exemplified at present in our local profession. In fact indirectly our Rochester idea will encourage men to completely limit themselves to their specialty and refuse all other practice whatever.

And finally let me consider the probable indirect effect upon the internist and the general practitioner. The man in general practice knows in what he should excel. It is constantly suggested to him by his patients, his journals, and by the papers that he hears even by those who are advanced specialists of various subjects. What we need today is medical diagnosis and medical prophylaxis and treatment. The day of the adviser and counsellor in medicine to the family and to the individual is not waning, but actually in a newer sense just dawning. The general practitioner should be a physiologist and a psychologist. He must help his patients to avoid diseases and to attain to greater efficiency in their lives. The internist will strive to be all this and in a technical sense become a more proficient consultant. In fact this great department of internal medicine offers the widest choice of opportunities for concentration of effort, such as diagnostic and therapeutic technic and the study and treatment of disturbances of all the most important organs and systems of our bodies. The real merit and esteem of our profession lies with the general practitioner and the internist. As these advance and perfect themselves, the whole profession will more nearly attain its purpose which is to efficiently serve the community.

(Read before the Monroe County Medical Society, at Rochester, N. Y., October 22nd, 1912.)

